# Retinal Vessel Diameters and Their Relationship with Cardiovascular Risk and All-Cause Mortality in the Inter99 Eye Study: A 15-Year Follow-Up

**DOI:** 10.1155/2016/6138659

**Published:** 2016-12-07

**Authors:** Dragana Drobnjak, Inger Christine Munch, Charlotte Glümer, Kristine Faerch, Line Kessel, Michael Larsen, Nina C. B. B. Veiby

**Affiliations:** ^1^Center of Eye Research, Department of Ophthalmology, Oslo University Hospital and University of Oslo, Oslo, Norway; ^2^Department of Ophthalmology, Zealand University Hospital, Roskilde, Denmark; ^3^Faculty of Health and Medical Sciences, University of Copenhagen, Copenhagen, Denmark; ^4^Research Center for Prevention and Health, Glostrup, Denmark; ^5^Steno Diabetes Center, Gentofte, Denmark; ^6^Department of Ophthalmology, Rigshospitalet, Glostrup, Denmark

## Abstract

*Purpose*. To describe associations between retinal vessel diameters and cardiovascular risk markers and mortality.* Methods*. The present study included 908 persons aged 30 to 60 years. Vessel diameters were expressed as central retinal venular equivalent (CRVE) and central retinal arteriolar equivalent (CRAE). Multiple linear regression analyses and Cox regression models were used.* Results*. Multiple linear regression analyses showed that narrower CRAE was associated with higher systolic blood pressure, age, and higher HDL cholesterol, whereas wider CRAE and CRVE were associated with smoking. Narrower CRVE was associated with higher HDL cholesterol. In an age-adjusted model, associations between wider CRVE and risk of ischemic heart disease were found (*P* < 0.001). Wider CRVE was associated with all-cause mortality (HR = 2.02, *P* = 0.033) in a model adjusted for age, gender, and blood pressure. However, the association was not statistically significant after additional adjustment for smoking.* Conclusions*. The associations between retinal vessel diameters and known cardiovascular risk factors were confirmed. All-cause mortality was not associated with retinal vessel diameters when adjusting for relevant confounders.

## 1. Background

Cardiovascular disease (CVD), including ischemic heart disease and stroke, is the most common cause of morbidity and mortality in the world [1]. Established modifiable risk factors for ischemic heart disease include tobacco smoking, a diet high in saturated fatty acids, physical inactivity, high cholesterol, high blood pressure, and high body mass index [2]. These are all risk factors associated with retinal microangiopathy found in epidemiological studies [3, 4] and in animal models [5]. The eye offers noninvasive direct access to the microvasculature and, through high-quality imaging and software-assisted grading methods, the retinal vessel may be evaluated with high reproducibility [6, 7]. A number of studies have shown that changes in retinal vessel diameter predict risk of cardiovascular disease (CVD) [8, 9] and stroke mortality [10, 11], independently of traditional risk factors. Despite these data, there is an incomplete understanding of the relationship between retinal vessel diameter and cardiovascular events with inconsistent findings in the literature [12].

Cardiovascular risk factors can lead to deaths that are not primarily classified as cardiovascular, such as chronic obstructive pulmonary disease, diabetes mellitus, dementia, or even cancer [13, 14]. It is relevant to study the role of microvasculature in noncardiovascular mortality, because of the established link between small vessels and diseases such as diabetes mellitus, dementia, and chronic obstructive pulmonary disease [11]. Subclinical cardiovascular pathology contributes to those deaths. It has been shown that decrease in risk factors was associated with the decrease in morbidity and mortality [15, 16].

The aim of this study is twofold: (1) to examine the associations between retinal vessel diameters and cardiovascular risk factors and (2) to assess the association between retinal vessel diameter and all-cause mortality. It is difficult to compare associations between retinal vessel diameter and mortality between studies because methods, study populations, length of follow-up, ethnicity, and mortality in the background population tend to differ across studies. This is the first study to examine such relationships in a Danish population-based cohort.

## 2. Methods

### 2.1. The Inter99 Study

#### 2.1.1. Study Population

The Inter99 study is a population-based nonpharmacological intervention study of cardiovascular and metabolic characteristics and lifestyle. The aim of the Inter99 study was to assess the effect on incidence of ischemic heart disease (IHD) after screening and repeatedly group-based nonpharmacological intervention on lifestyle. An age- and sex-stratified random sample of 13,016 individuals from seven birth cohorts (years of birth 1939-40, 1944-45, 1949-50, 1954-55, 1959-60, 1964-65, and 1969-70) living in 11 municipalities of the south-western part of Copenhagen County on December 2nd 1998 were invited to participate, of which 6906 turned up for the investigation. Of these, 122 were excluded due to either alcoholism or drug abuse or because of linguistic problems; thus 6784 subjects were included in the study. The study participants were drawn from the Danish Civil Registration System in which a unique 10-digit number registers all inhabitants in Denmark, making linkage across time and registers accurate.

#### 2.1.2. Study Procedures

All participants went through a screening program at the Research Centre for Prevention and Health at Glostrup University Hospital, Copenhagen, Denmark. Prior to the examination the participants had been fasting for ≥8 hours. All had their blood pressure (BP) measured twice with a mercury sphygmomanometer after 5 min of rest in lying position. Height and weight were measured without shoes, and body mass index (BMI) was calculated (kg/m^2^). Waist and hip circumference was measured in cm and waist/hip ratio was calculated. Fasting blood samples were drawn for assessment of total cholesterol, HDL cholesterol, and triglycerides. VLDL and LDL were calculated by Friedewald's equation. An oral glucose tolerance test (OGTT) was performed. If systolic BP was above 140, it was repeated twice. Smoking was assessed as number of pack years ever smoked. One pack year equals 20 cigarettes per day for one year. History of lifestyle, education and profession, chronic diseases, family history of chronic diseases, health care system contacts, and lifestyle consciousness was obtained from questionnaire. A detailed description of The Inter99 study has previously been published [17].

Information on age, sex, height, familial occurrence of acute IHD, previous ischemic disease, diabetes, systolic BP, cholesterol (incl. HDL), weight, smoking, and diabetes was entered into the PRECARD program to assess an individuals' 10-year risk of fatal and nonfatal ischemic heart disease. Each person was simulated in the computer program as 60 years old to be able to compare risk among different age groups [18]. The PRECARD program contains a new coronary risk score (the Copenhagen Risk Score) for myocardial infarction. The PRECARD and the Copenhagen Risk Score was described in detail previously [19].

### 2.2. The Inter99 Eye Study

#### 2.2.1. Study Population

A subgroup of 1437 persons was chosen based on the following criteria: (1) an age- and sex-stratified control group was randomly polled to match the background population and (2) persons with a high risk of IHD, type 2 diabetes mellitus diagnosed by the oral glucose tolerance test, known diabetes mellitus, or impaired glucose tolerance. A total of 970 persons (67.5%) underwent the eye examination.

Individuals were considered as high risk if they either had an absolute risk in the upper quintile of the distribution (according to the PRECARD classification) stratified according to age and sex or had one or more of the following risk factors: daily smokers (one or more grams of tobacco daily), systolic blood pressure of 160 or more (the lowest value of at least three measurements) or in antihypertensive treatment, total cholesterol of 7.5 mmol/L or more, body mass index of 30 or more, history of diabetes, or had diabetes or impaired glucose tolerance as evaluated from the OGTT [19]. Persons without fundus photographs of acceptable quality (*n* = 62) were excluded, leaving 908 persons available for analysis. Excluded participants did not differ from the included (parameters from Table 1 were tested). Only information from right eye was used, because large epidemiological studies have demonstrated that the correlation of computer-assisted measurements is high between the two eyes for CRAE and CRVE and that measurement from only one eye can adequately represent the retinal vessel diameters [20, 21]. If the retinal photographs from the right eye were not gradable, photographs from the left eye were used.

#### 2.2.2. Study Procedures

Study participants were asked about present or past history of ophthalmic disorders, surgery, ocular medication, and family history of cataract and glaucoma. The ophthalmic examination included a determination of visual acuity, fundus photography (Trc-50X camera; Topcon Corp. Tokyo, Japan; with 1024 × 1024 pixel CV-1000 back-piece, AngioVision 1000; MediVision, Yokneam Illit, Israel), and ophthalmoscopy. Image analysis was made of 60-degree digital grey-scale (red-free) fundus photographs centred on the optic disc or macula. A green filter for red-free photographs was used to enhance the sharpness and contrast of the blood column in the vessels. Retinal vessel diameters were measured using a Danish custom-developed semiautomatic computer algorithm [22]. According to international standards (Vessel Measurement System, IVAN protocol, version 2, University of Wisconsin) [23], retinal vessel diameters were measured in 60° digital grey-scale (red-free) fundus photographs. We tested the IVAN software against our own custom-developed software in twenty red-free fundus photographs. Absolute distances were calculated assuming a uniform vertical optic disc diameter of 1800 *µ*m. A standard grid containing three concentric circles was placed on the images. The inner circle demarcated an average optic disc (circle was placed manually), the middle circle demarcated 0.5 disc diameters (DD) from the outer rim of the optic disc, and the outer circle demarcated 1.0 DD from the outer rim of the optic disc. The program identified the six largest arteries and the six largest veins and calculated the central retinal artery equivalent (CRAE) and the central retinal vein equivalent (CRVE) according to the formulas described by Knudtson et al. [24]. The grader then identified arteries and veins between the outer two circles, using red and blue lines, respectively, to delineate the borders of the blood vessels, which were defined, in this context, as the edges of the blood column the vessel wall being translucent. Vessel diameters were expressed as CRAE and CRVE and were measured in micrometres [25] at the baseline examination.

### 2.3. Follow-Up of Study Participants

#### 2.3.1. Registers

The participants' unique personal identification number was linked to the Danish Civil Registration System, to the National Patient Registry, and to the Danish Register of Causes of Death for vital status and cause of death. IHD was defined as either admission to hospital (inpatient or outpatient) or causes of death with ICD-8 codes 410–414 and ICD-10 codes I20–I25 or surgery codes 300.09–304.99 and KFNC-KFNH (since 1996; bypass, recanalization, or reconstruction of coronary arteries). Stroke events were defined as admission to hospital (inpatient or outpatient) or causes of death with ICD-8 codes 430–434 + 436 and ICD-10 codes I60–I64 + I69. The following codes covered noncardiovascular mortality: C00–C97 (cancer), F00–F03 (dementia), J15–J19 (pneumonia), J30–J98 (chronic respiratory disease), or K72 (acute and subacute hepatic failure) and the remaining codes (other).

Participants were followed up from date of study entry until date of death, up till the 31st of December 2013.

## 3. Statistics

The PRECARD values are expressed in % unit and were not normally distributed. We therefore transformed the PRECARD values with the natural logarithm (ln).

Associations of retinal vessel diameters with the estimated 10-year absolute risk of IHD at 60 years (PRECARD score) were analyzed using univariate linear regression analyses. Relations between retinal vessel diameters and individual risk factors for IHD were analyzed using multiple linear regression. First, each risk factor was modeled separately to analyze the relationship of each risk factor with CRAE and CRVE. When high colinearity was found for an independent variable in a regression model with several independent variables (Variance Inflation Factor > 5), this variable was removed from the regression model. We used multiple linear regression analyses with backward variable selection. Step 1 was to include all the variables that were significant in unadjusted analyses (age, systolic blood pressure, total cholesterol, HDL cholesterol, smoking, diabetes, HbA1c, fasting p-glucose, 2-hour OGTT p-glucose, BMI, and weight). Step 2 was to remove the variable with the highest *P* value and to run the model once more. Step 3 was to repeat step 2 until all the remaining variables were significant.

Vessel diameters were chosen as dependent variables, and the estimated 10-year risk of ischemic heart disease (IHD) and individual risk factors for IHD were chosen as independent variables.

Multivariate Cox regression analyses were used to determine the association of retinal vessel diameters with all-cause mortality. Estimates were presented as hazard ratios (HRs) (95% confidence intervals) after adjustment for confounders (age, gender, blood pressure, and smoking).

There were no significant interactions between the two ascertainment groups. This means that the associations between CRAE and CRVE and cardiovascular risk factors were similar in the two ascertainment groups. Therefore, we presented the results of the total population of 908 persons.

The level of statistical significance was set at *P* = 0.05. Statistical procedures were performed using the SPSS software (version 18.0).

## 4. Results

The clinical characteristics of the participants are presented in Table 1. The mean (SD) age at baseline examination was 48 (8) years. The study included 48% men and 52% women. Twenty-three percent of participants had diabetes mellitus and 8% were current smokers. Vessel diameters were normally distributed. Mean (SD) CRAE was 163.3 (15.8) *µ*m and CRVE was 251.0 (20.9) *µ*m (Table 1).

When we tested the IVAN software against our Danish custom-developed semiautomatic software in twenty red-free fundus photographs, we found an intraclass correlation coefficient (ICC) of 0.8 (95% CI: 0.5 to 0.9) for CRAE and 0.9 (95% CI: 0.8 to 0.9) for CRVE. Furthermore, when we tested the IVAN software against our Danish custom-developed software, there were no statistically significant differences between measurements of CRAE (*P* = 0.448) and CRVE (*P* = 0.828).

The intergrader reliability (ICC) between 2 independent masked graders using the Danish custom-developed software was 0.9 for arteries and 0.8 for veins, measured on 45 right eyes. The mean difference in vessel diameter among the two graders was 3.9 *µ*m (0.024%) for arteries and 6.2 *µ*m (0.025%) for veins.

### 4.1. Retinal Vessel Diameter and Cardiovascular Risk Factors

#### 4.1.1. Central Retinal Artery Equivalent (CRAE)

When adjusting for age no association between CRAE and IHD risk was found (Table 2). Multiple linear regression analyses showed that smokers had wider CRAE than nonsmokers and that CRAE decreased with increasing age, increasing HDL cholesterol and increasing systolic blood pressure (Table 3).

#### 4.1.2. Central Retinal Vein Equivalent (CRVE)

When adjusting for age a significant association between wider CRVE and IHD risk was found, *P* < 0.001 (Table 2).

Multiple linear regression analyses showed that smokers had wider CRVE than nonsmokers and that CRVE decreased with increasing HDL (Table 3).

### 4.2. Retinal Vessel Diameter and Mortality

Of the 61 participants who died, 12 persons died of CVD (8 from IHD and 5 from stroke) and 49 died from other causes. Of the participants who died, 54.0% were men and 46.0% were women. The mean (SD) age at the time of death was 58 (6) years for participants who died of CVD causes and 60 (7) years for participants who died from other causes.

Noncardiovascular cause of death in 49 participants (*n* = 49) was due to cancer (*n* = 28), dementia (*n* = 4), chronic respiratory disease (*n* = 2), acute and subacute hepatic failure (*n* = 1), and/or unknown causes (*n* = 14).

There was no significant difference in CRAE (*P* = 0.324) and CRVE (*P* = 0.954) between participants who died from CVD compared with other causes of death (data not tabulated).

#### 4.2.1. Central Retinal Artery Equivalent (CRAE)

All-cause-mortality was not significantly associated with CRAE with a HR of 0.75 (95% CI: 0.3 to 1.5) (*P* = 0.431), in a model adjusted for age, gender, blood pressure, and smoking (Table 4).

Cardiovascular mortality was not significantly associated with CRAE with a HR of 0.57 (95% CI: 0.1 to 2.5) (*P* = 0.465), in a model adjusted for age, gender, blood pressure, and smoking (data not tabulated).

#### 4.2.2. Central Retinal Vein Equivalent (CRVE)

When the third tertile of CRVE was compared to the first tertile of CRVE, all-cause mortality was significantly associated with higher CRVE with a HR of 2.02 (95% CI: 1.0 to 3.8, *P* = 0.033), in a model adjusted for age, gender, and blood pressure. When additionally adjusting for smoking it was no longer significant with a HR of 1.34 (95% CI: 0.6 to 2.6, *P* = 0.392) (Table 4).

Cardiovascular mortality was not significantly associated with CRVE with a HR of 1.06 (95% CI: 0.2 to 5.0) (*P* = 0.933), in a model adjusted for age, gender, blood pressure, and smoking (data not tabulated).

## 5. Discussion

In this large general population study of adult Danes, we confirmed the classical associations between retinal vessel diameters and known cardiovascular risk factors. Furthermore, we found an association between wider CRVE and calculated risk of IHD. We did not find an association between retinal vessel diameters and all-cause mortality.

Higher serum HDL cholesterol was independently associated with CRAE narrowing and CRVE narrowing in our study, which is consistent with findings from some [26, 27] but not all studies [7, 28]. However, clinical studies have not presented a consistent pattern of association between retinal vessel diameter and dyslipidemia [29]. Some genetic mechanisms that raise plasma HDL cholesterol do not seem to lower risk of myocardial infarction [30]. Thus, it is suggested that more may be learned from a closer inspection of those mechanisms. In our study wider CRAE and CRVE were associated with current smoking which was also reported previously [27]. Mechanisms behind these associations remain unclear, but smoking-induced increase in nitrous oxide production, potassium channel activation [5], and possible tissue degeneration might explain the association with a wider CRVE [31–34]. In our cohort we confirmed an association between narrowed CRAE and aging as well as between narrowed CRAE and high blood pressure [27, 35]. In our study, which used the PRECARD program based on the Copenhagen Risk Score, higher CRVE was associated with IHD risk, which is in accordance with findings from previous studies [9]. Thus, the data from the study suggest a single fundus photograph cannot yet be used in the evaluation of an individual patient's risk of cardiovascular disease.

It has been hypothesized that changes in the retinal vessel diameters might be markers for general health and not necessarily for cardiovascular health only [11]. In 1978, in a study of 50-year-old men, Svardsudd et al. found that focal arteriolar narrowing was associated with increased 12-year all-cause and cardiovascular mortality rates after controlling for systolic blood pressure and other risk factors [36]. An unexpected association with cancer and other noncardiovascular related mortality was also found. In February 2016, Mutlu et al. showed that narrower retinal arteriolar diameters and wider retinal venular diameters were associated with all-cause mortality [11]. They followed 5674 persons from an adult Dutch population during 25 years, where 3794 persons died, 1034 of those from cardiovascular causes. While narrower arterioles were associated with cardiovascular mortality, wider venules were equally associated with cardiovascular and noncardiovascular mortality [11]. In our study, we compared if all-cause mortality risk was increased in patients who have the widest vs thinnest vessels. Larger CRVE explained the relationship between retinal vessel diameters and all-cause mortality, but this relationship was no longer significant after additional adjustment for smoking. Adjustment for smoking reduced the estimated difference between the highest tertile compared to the lowest tertile of retinal venular caliber by 34%. It is reasonable to think that the results represent a cofounding effect of smoking in the investigated association between retinal vascular diameters and all-cause mortality. Other factors explaining the lack of association may be the relatively short follow-up time and the low number of deaths.

Diseases as chronic obstructive pulmonary disease, dementia, and even cancer, which are primarily classified as noncardiovascular diseases, may have common risk factors and a partly vascular pathogenesis. Still, most population-based cardiovascular studies are focused on large vessels such as aorta or carotid arteries [37], despite evidence that microcirculation is important for cardiovascular health as well [38]. In our cohort, all-cause mortality was not associated with retinal vessel diameters. Since studies of past smokers suggest that the impact of smoking on CRVE is reversible [39], it remains for our results to be proven in a larger study population.

In several large population-based epidemiological studies, IVAN software has demonstrated substantial reproducibility (intergrader correlation coefficient ranged from 0.6 to 0.9) [29]. The ICC results in our study indicate that retinal vessel diameters calculated by the Danish custom-developed semiautomatic software are comparable with retinal vessel diameters calculated by the IVAN software and have a high interrate reliability. The lengths of 95% confidence intervals for the calculated ICC's are relatively short. Therefore, 20 pictures are significant for calculating these ICCs. Thus, the Danish custom-developed software provides a reliable research tool for objective assessment of structural vascular changes.

The study was designed to optimize fundus vessel imaging by applying direct digital imaging in red-free illumination rather than digitization of colour slides as used in earlier studies [40, 41]. Strengths of the study include the population-based sample. Limitations include the absence of correction for refraction and electrocardiographic synchronization of the fundus camera to the cardiac cycle which may influence the vessel calibers [42] and the low number of deaths. Furthermore, calibration of the computer-assisted program is crucial when determining the true size of a fundus feature. Studies published in the 1990s showed that the true value of one standard vertical disk diameter was equivalent to 1800–1900 *µ*m [43]; later it was standardized to 1800 *µ*m. This is now accepted as a reference for calibration to compensate for the effect of camera magnification on the vessel caliber measurement in the computer-assisted programs [29]. This method potentially introduces bias, since individuals have different size optic discs, refraction, and axial length. Researchers within the field have accepted the magnification problem as bias, for which there is no good solution at the moment.

In conclusion, we confirmed the classical associations between retinal vessel diameters and known cardiovascular risk factors, and we found that larger CRVE is associated with increased IHD risk. Furthermore, we did not find an association between retinal vessel diameters and all-cause mortality after adjusting for relevant confounders. However, given the heterogeneity of previous studies which differ in methods, populations, ethnicity, and length of follow-up, it remains for our results to be proven in a larger study population. Longer follow-up time may reveal associations that were not demonstrable at ages 30–60 years, after 15 years of follow-up and with a high probability of survival.

## Figures and Tables

**Table 1:** Clinical characteristics of the study population.

Number of individuals	Individuals included *n* = 908	Individuals excluded *n* = 62
Age (years)	48 (8.0)	48 (8.4)
Males/females	442/466 (48%/52%)	28/34 (45%/55%)
NGT/IGT/DM^*∗*^	473/206/199 (54%/23%/23%)	37/10/12 (63%/17%/20%)
Smoker	282/339/209/71	26/14/17/4
(never, occasionally, ex, present)	(31%/38%/23%/8%)	(43%/23%/28%/6%)
Smoking (number of pack years)^*∗∗*^	5.0 (0.0–112.5)	8.0 (0.0–52)
Previous AMI (yes/no)	121/787 (13%/87%)	5/57 (8%/92%)
Family history of CVD (yes/no)	21/887 (2%/98%)	0/62 (0/199%)
HbAIc (mmol/mol)^*∗∗*^	5.9 (4.1–14.4)	5.9 (5.0–8.4)
Fasting p-glucose (mmol/L)^*∗∗*^	5.6 (3.0–20.9)	5.7 (4.4–14.4)
2-hour OGTT p-glucose (mmol/L)^*∗∗*^	6.9 (2.3–30.6)	6.4 (4.0–17.3)
Total cholesterol (mmol/L)	5.8 (1.1)	5.9 (1.2)
HDL cholesterol (mmol/L)	1.4 (0.4)	1.4 (0.43)
Triglycerides (mol/L)	1.7 (2.4)	1.5 (1.0)
LDL cholesterol (mmol/L)	3.6 (1.0)	3.8 (1.0)
BMI (kg/m^2^)^*∗∗*^	25.7 (17.5–53.4)	27.5 (17.6–45.9)
Waist circumference (cm)	103.4 (11.0)	102.6 (10.6)
Systolic blood pressure (mmHg)	136.0 (19.3)	139.1 (20.2)
Diastolic blood pressure (mmHg)	85.6 (12.0)	87.2 (13.2)
Central retinal artery equivalent diameter (CRAE) (*µ*m)	163.3 (15.8)	170.3 (19.3)
Central retinal vein equivalent diameter (CRVE) (*µ*m)	251.0 (20.9)	260.8 (25.5)

^*∗*^NGT: normal glucose tolerance, IGT: impaired glucose tolerance, and DM: diabetes mellitus (known and newly diagnosed).

Values are given as means (SD) unless otherwise stated.

^*∗∗*^Values are given as medians (interquartile range).

**(a):** 

Total number *N* = 908	Central retinal artery equivalent diameter (CRAE), *µ*m		
	Coefficient (*µ*m)	95% confidence interval	*P* value
Risk of ischaemic heart disease (IHD)%	−0.2	−1.5 to 1.0	0.685

**(b):** 

	Central retinal vein equivalent diameter (CRVE), *µ*m		
Risk of ischaemic heart disease (IHD)%	3.3	1.5 to 5.0	<0.001

**(a):** 

Total number *N* = 908	Central retinal artery equivalent diameter (CRAE), *µ*m			
Risk factor	Increment	Coefficient (*µ*m)	95% confidence interval	*P* value
Systolic BP (mmHg)	1 mmHg	−0.2	−0.3 to −0.2	<0.001
Age (*µ*m/1 year)	1 year	−0.2	−0.4 to −0.1	0.002
Smoking (yes/no)	Smokers versus nonsmokers	4.0	1.9 to 5.9	<0.001
HDL (mmol/L)	1 mmol/L	−3.8	−6.2 to −1.3	0.003

**(b):** 

	Central retinal vein equivalent diameter (CRVE), *µ*m			
Smoking (yes/no)	Smokers versus nonsmokers	9.0	6.3 to 11.8	<0.001
HDL (mmol/L)	1 mmol/L	−6.8	−10.1 to −3.5	<0.001

The model initially included the following variables: age, gender, total cholesterol, HDL cholesterol, LDL cholesterol, triglycerides, systolic blood pressure, smoking, body mass index, diabetes mellitus, family history of cardiovascular disease, and previous heart disease.

**(a):** 

By tertiles (ranges in *μ*m)	Total number *N* = 908	Central retinal artery equivalent diameter (CRAE), *µ*m			
	Number of participants who died *N* = 61	^*∗*^Model 1: hazard ratio (95% CI)	*P* value	^*∗∗*^Model 2: hazard ratio (95% CI)	*P* value
1st ≤ 156.6	21	1.0 (reference)		1.0 (reference)	
2nd 156.6–169.8	25	1.39 (0.7 to 2.5)	0.280	1.34 (0.7 to 2.4)	0.338
3rd ≥ 169.8	15	0.86 (0.4 to 1.7)	0.680	0.75 (0.3 to 1.5)	0.431

**(b):** 

		Central retinal vein equivalent diameter (CRVE), *µ*m			
1st ≤ 242.0	14	1.0 (reference)		1.0 (reference)	
2nd 242.0–260.2	21	1.50 (0.7 to 2.9)	0.245	1.35 (0.6 to 2.6)	0.390
3rd ≥ 260.2	26	2.02 (1.0 to 3.8)	0.033	1.34 (0.6 to 2.6)	0.392

^*∗*^Model 1 (adjusted for age, gender, and BP) and ^*∗∗*^Model 2 (adjusted for age, gender, BP, and smoking).

## List of Abbreviations

CRVE: Central retinal vein equivalent diameter
CRAE: Central retinal artery equivalent diameter
AVR: Arteriole-to-venule ratio
IHD: Ischemic heart disease
CVD: Cardiovascular disease.
